# Innovative Soil Management and Micro-Climate Modulation for Saving Water in Peach Orchards

**DOI:** 10.3389/fpls.2020.01052

**Published:** 2020-07-22

**Authors:** Pasquale Campi, Liliana Gaeta, Marcello Mastrorilli, Pasquale Losciale

**Affiliations:** ^1^ Research Centre for Agriculture and Environment, CREA—Council for Agricultural Research and Economics, Bari, Italy; ^2^ Department of Soil, Plant and Food Sciences, University of Bari “Aldo Moro”, Bari, Italy

**Keywords:** deficit irrigation, soil stress coefficient, irrigation water productivity, mulching, shading hail net

## Abstract

Microclimatic and soil management studies emphasize that roofing above the canopy or soil mulching contributes to reduce water losses from horticultural cropping systems and, at the same time, to increase water use efficiency. The aim of this 2-year on-farm study, carried out on a late ripening peach (cv. California) orchard, was to investigate the combined effect of water supply (full or deficit irrigation, DI), incoming light (hail or shading net), and soil management (tilling or mulching) on: microclimate; fruit growth; yield; irrigation water use productivity (WP_I_); and soil water stress coefficient (Ks). Shading hail net reduced air temperature (−1°C), wind speed (−57%), solar radiation (−32%), while increased relative air humidity (+9.5%). Compared to the control treatment (hail net coverage, soil tillage, and full irrigation), the innovative management (DI + shading hail net + mulching) reduced seasonal volumes of irrigation water (−25%) and increased both final yield (+36%) and WP_I_ (+53%). Saving water resources without losing yield is an achievable goal by peach orchards growing under the Mediterranean climate if the DI agro-technique is adopted conjointly with shading hail net and soil mulching.

## Introduction

Deficit irrigation (DI) is a sustainable way to use the water resources in the Mediterranean cropping systems. Results from the scientific literature on DI allow concluding that it is possible to reduce irrigation volumes without penalizing yields (Fereres and Soriano, 2007; Ruiz-Sánchez et al., 2010; Ripoll et al., 2014; Maatallah et al., 2015). In addition, in the case of tree cultivation systems, two additional advantages can be achieved through DI: improvement of fruit quality in terms of higher SSC (soluble solid content) and control of tree vigor (Crisosto et al., 1994; Behboudian and Mills, 1997; Mpelasoka et al., 2000; Faci et al., 2014; Guizani et al., 2019). Finally, the economic and agronomic benefits of DI are coupled with the environmental ones (Villalobos and Fereres, 2016).

Climate scenarios in the Mediterranean region, both in the short and medium terms, stress the importance of DI, as well as of tuning the set of water-saving agronomic techniques accordingly (Allen et al., 2018).

Screen covers placed over the orchard canopies modify microclimate (Tanny et al., 2009; Bastias et al., 2011; Losciale et al., 2011; Tanny et al., 2014) since they affect radiation, wind, temperature, and humidity. If adequately managed, the canopy covering regulates the atmosphere water demand (Pirkner et al., 2014) in order to reduce water to be supplied through irrigation (Rana et al., 2004; Lopez et al., 2018). Orchard covering by shading hail net has been proposed as a technique to improve tree water status and water use efficiency where water is scarce (Nicolas et al., 2005; Corelli-Grappadelli and Lakso, 2007; Losciale et al., 2011; Abouatallah et al., 2012; Girona et al., 2012; Lopez et al., 2018).

Mulching materials placed over the soil surface reduce soil evaporation by cutting off the vapor transport path between the soil surface and the atmosphere and contrasting weeds growth and the related transpiration as well as. Compared to soil tillage, mulching has significant advantages in soil moisture preservation (Kader et al., 2017), reducing soil evaporation losses and improving crop yields (Taparauskiene and Miseckaite, 2014; Jia et al., 2018; Yu et al., 2018).

Although some researches have analyzed the effects of shading hail net (Corelli-Grappadelli and Lakso, 2007; Losciale et al., 2011; Abouatallah et al., 2012; Esmail et al., 2017; Lopez et al., 2018) or of mulching (Wang H. et al., 2015; Wang C. et al., 2015; Lepaja et al., 2016) on the relationship between yield and soil water stress, no study has been designed expressly to determine the suitability of shading hail net in addition to mulching in attenuating the water stress induced by the DI technique at the soil level.

Since DI does cause soil water stress, a research prerequisite consists in handling at the field scale a friendly indicator to define the stress experienced by the crop along the whole season, in place of the soil stress coefficient (Ks) which fluctuates during the vegetative cycle at the daily scale, following the soil water balance variations. Successively the combined effects of light screening and soil management on the Ks seasonal indicator can be analyzed, as well as the relationships between such synthetic Ks and plant behavior.

The aim of the study was to verify the hypothesis that canopy and soil coverages acting together can alleviate soil water stress when irrigation is supplied with limited volumes. A seasonal indicator for the Ks variations is proposed as a deputy criterion for evaluating the effects of the adopted agro-techniques on the peach orchard performances (fruit growth and yield).

Conclusions from this study will provide friendly tools for designing the suitable DI agronomic strategies which reduce the impact on yield and on water resources of horticultural species growing in areas characterized by the Mediterranean climate.

## Materials and Methods

### Experimental Site

The study was conducted during the 2017 and 2018 seasons in southern Italy (Cerignola, lat: 41° 20′, long: 15° 56′, altitude 40 m a.s.l.), in a private farm. Observations were carried out on a 15-year old peach orchard of a late ripening cv (California), trained as open vase and grafted on GF677 rootstock, spaced 5.0 m (between the rows) × 3.0 m (within the row), and managed according to the usual farm practices. The peach experimental set-up was realized on a 3,600 m^2^ flat surface, within a 5 ha commercial orchard. The physico-chemical characteristics were similar within the experimental plot (**Table 1**). Soil texture was classified as clay-loam (Soil Survey Staff, 1975) having 314 g kg^−1^, 375 g kg^−1^ and 311 g kg^−1^ of sand, silt, and clay, respectively, determined by the hydrometer method. Soil water content in volume at field capacity (fc, −0.03 MPa) and wilting point (wp, −1.5 MPa) were 0.32 and 0.18 m^3^ m^−3^, respectively (measured in the Richards chambers). The soil water reserve was moderate (192 mm) because the root system did not develop below 0.6 m in this site.

**Table 1:** Main physical–chemical characteristics of the soil sampled within the experimental site.

Parameter	average	± sd
Sand (g kg^−1^)	314	16
Silt (g kg^−1^)	375	27
Clay (g kg^−1^)	311	29
E.C. (dS m^−1^)	0.7	0.06
Field Capacity (m^3^ m^-3^)	0.32	0.04
Wilting Point (m^3^ m^−3^)	0.18	0.03
SOC (g kg^−1^)	10	1.5
Total N (g kg^−1^)	0.8	0.3
Available P (mg kg^−1^)	26	1.7
Exchangeable K (mg kg^−1^)	257	41

Each value is the average of 24 replicates (s.d. = standard deviation).

The experimental site is under the Mediterranean climate, characterized by warm and dry summers, with minimum and maximum annual air temperatures ranging from 0 to 5°C and 32 to 43°C, respectively. The annual rainfall is 560 mm. Rains are distributed mainly in autumn and late winter, and they are negligible in the spring–summer period (Campi et al., 2012). Therefore, most species can be cultivated successfully only by supplying irrigation water (Campi et al., 2016).

The input agrometeorological data (daily rainfall, minimum and maximum temperatures, relative air humidity, solar radiation, wind speed) for calculating reference evapotranspiration according to Allen et al. (1998) were provided by an agrometeorological station located at a short distance from the experimental site. Following the Allen et al. (1998) methodology, irrigations were scheduled whenever, in the soil layers colonized by the root system, the readily available water (RAW) was completely depleted. To perform the soil water balance in the peach orchard, the retained values for crop coefficients (Kc_ini_ = 0.15; Kcm_ed_ = 0.80; Kc_end_ = 0.60) and depletion fraction (p = 0.5) were those tabulated in the 56 FAO handbook. Corrections of Kc_ini_ (for precipitation events), Kc_med_, and Kc_end_ (for climatic conditions and crop height) were performed according the Allen et al. (1998) methodology.

Water was supplied by a drip irrigation system having two drippers *per* tree and a flow rate of 8 l h^−1^ *per* dripper. Seasonal irrigation volumes, irrigation depth (mm), and number of irrigations *per* season (n.) scheduled in full (FI) and deficit irrigation (DI) treatments are reported in **Table 2**.

**Table 2:** – Irrigation seasonal volume (I, mm), depth (mm), and number (n.) and Deficit Irrigation Ratio (DIR = I(DI)/I(FI)) observed in two treatments (FI and DI) and two seasons (1^st^ June–10^th^ September).

Season	FI			DI			DIR
	I (mm)	depth (mm)	n.	I (mm)	depth (mm)	n.	
2017	477	25.1	19	358	18.8	19	0.75
2018	405	28.9	14	304	21.7	14	0.75

### Crop Management and Treatments

**Figure 1:**
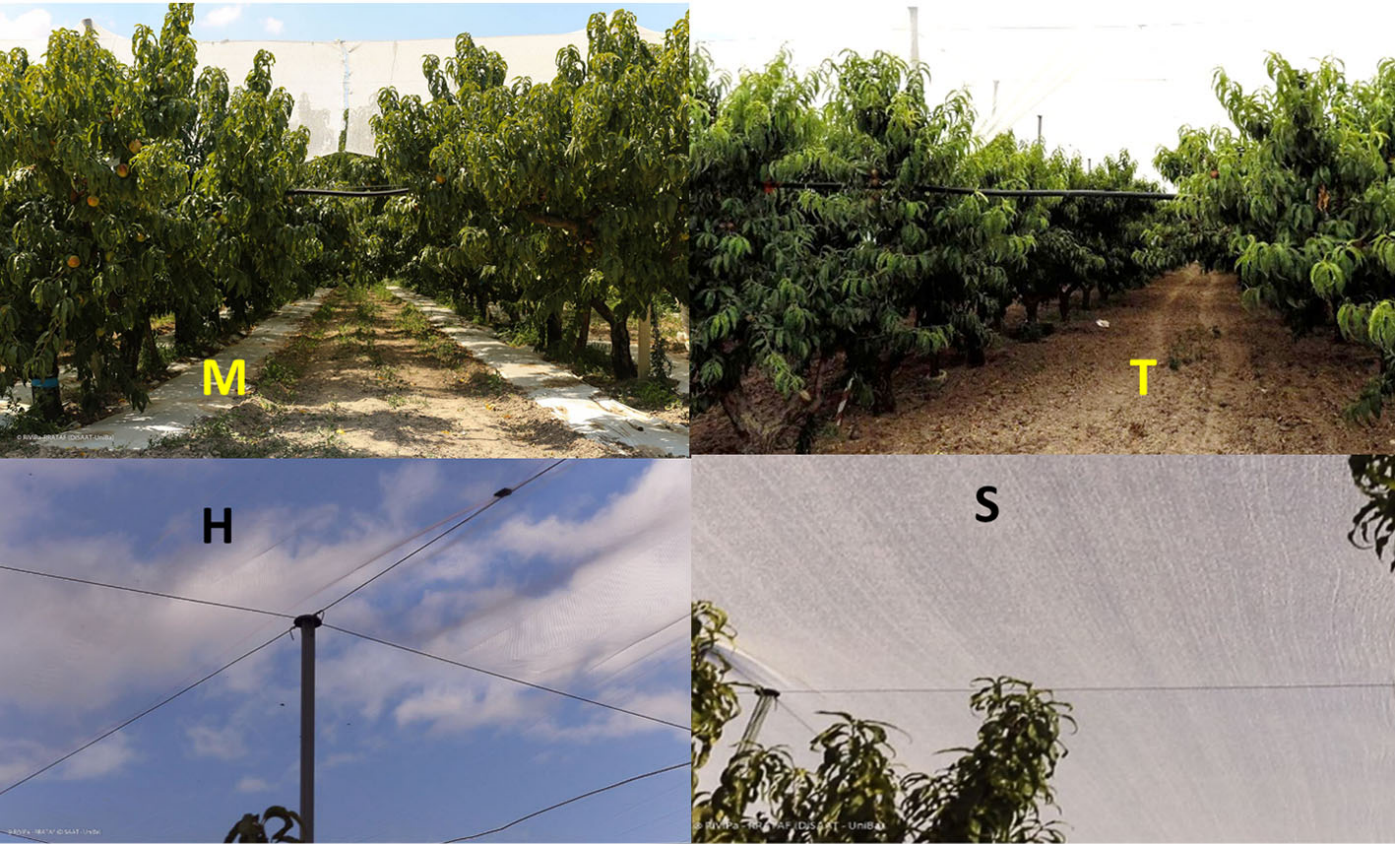
Soil management (T, tillage; M, mulching) and vegetation roofing (H, ail net; S, shading net) at the experimental site.

Three agromanagement factors (**Figure 1**) were retained:

- two light regimes (Lr): reduction of incident solar radiation by 10% for hail nets (H) and by 30% for shading hail nets (S), both having high diffusivity. The nets were placed horizontally over the trees (4 m above soil surface) to shade all the rows within the test area. The nets were installed using a supporting tensile structure with longitudinal and transversal steel cables tensioned to a supporting structure of concrete and wood as described by Castellano et al. (2008).
- two soil managements (Sm): tilled soil (T) and mulching with a biodegradable film (M) on the row. The M treatment was realized by laying the film tape (2 m wide) at the beginning of each vegetative season along each side of the rows; while in autumn (after harvest) it was ploughed into the soil. The biodegradable film had a black surface facing the soil and a white surface facing the sky to reflect the incident radiation.
- two irrigation regimes (Ir): full irrigation (FI), supplying the amount of water lost by evapotranspiration (ETc) and deficit irrigation (DI), restoring 50% of ETc (from August until the end of irrigation season) in 2017 or 75% of ETc during the whole irrigation season (1/6–10/9) in 2018. In average, the seasonal deficit imposed for both the years was 25% (**Table 2**).

Each Lr treatment (H and S) was sized (25 m × 60 m) in a way that the central part of the surveyed plots was not influenced by the diffuse light coming from the nearby plots having different Lr. Moreover, the size of each plot allowed detecting the differences between the micro-climatic conditions monitored in H and S treatments.

**Figure 2:**
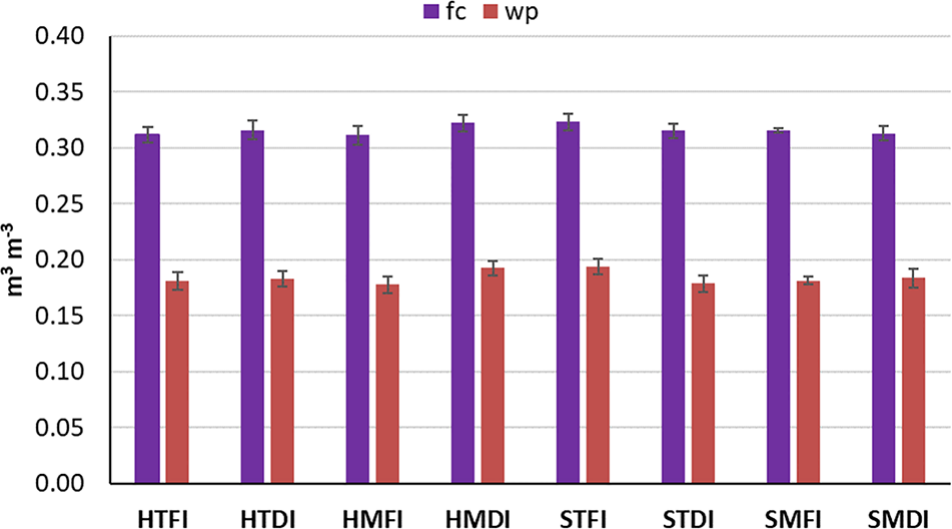
Field capacity (fc) and wilting point (wp) detected in each treatment. Each bar represents the average of 3 samples + s.e. No letter indicates no significant differences according to Duncan test (P < 0.05).

Within each light regime plot, soil managements (T, M) and irrigation regimes treatments (FI, DI), were set-up orthogonally each other. The distances between the irrigation regime treatments were enough to avoid the horizontal flow of water between adjacent plot treatments. The great surfaces needed to manage these kind of factors (light, soil, water) under field conditions do not allow the complete randomization of the treatments; however, soil properties showed a low variability which get realistic to retain uniform the experimental site (**Table 1**, **Figure 2**).

The combination of the three experimental factors (Lr, Sm, and Ir) resulted in eight different crop management treatments: HTFI (control), HTDI, HMFI, HMDI, STFI, STDI, SMFI, and SMDI. To Each treatment corresponded a surveyed area of 450 m^2^ where 30 trees were growing.

### Field Observations

During the irrigation season (June–September) and for H and S managements, the climatic parameters: air temperature (Tair), relative humidity (RH), wind speed (u) and solar radiation (Rs), were monitored by agrometeorological sensors placed at 0.5 m above the canopy and below the H and S shelters.

Soil water content (SWC) in volume was measured by capacitive probes (10HS, Decagon Devices Inc., USA). For each treatment, three points were monitored (Padilla-Dìaz et al., 2016). At each point, three capacitive probes were installed horizontally into the soil profile and transversely to the row, at −0.1, −0.3, and −0.5 m from the soil surface in order to intercept the dynamics of SWC below the dripping lines. All sensors were connected to data-loggers (Winet srl, Italy), and data were transferred on a web-server *via* GPRS mode. Soil specific calibration functions were used to calculate the volumetric SWC.

Integrated soil–water content on a daily basis (SWC_i_) was determined for the soil profile (0.6 m) by integrating the values measured at each depth, since each probe was supposed to detect the water content in a 0.2 m soil layer (Campi et al., 2019):

(1) $$\int_{0}^{0.6}SWC_{i}=SWC_{i(-0.1)}(m^{3}m^{-3})\cdot 0.2(m)+SWC_{i(-0.3)}(m^{3}m^{-3})\cdot 0.2(m)+SWC_{i(-0.5)}(m^{3}m^{-3})\cdot 0.2(m)$$

The SWC_i_ measurements from the three points were pooled in order to obtain a single average value for each of the eight orchard managements.

In order to evaluate the effect of the different orchard management on soil as well as on tree performance, three trees similar for dimension vigor and health state, in correspondence with the soil moisture probes, were chosen within each treatment. As reported in other studies, three trees per treatment, considering each tree as a replicate, could be considered representative in uniform experimental sites (Abou Kheira and Atta, 2009; Kassem et al., 2011; Ballester et al., 2013). Fruit growth was monitored for both years on 12 fruits per tree, assuming the fruit shape as a spheroid and measuring periodically the three diameters to calculate its volume (cm^3^) and the related Absolute Growth Rate (AGR, cm^3^ d^−1^) of fruit volume (Pérez-Pérez et al., 2014). Potential yield (t ha^−1^) was evaluated measuring the fruit weight *per* tree (kg tree^−1^), the related number of fruits (n. tree^−1^), and the average fruit weight (g). Harvest was performed when fruit achieved their commercial ripening checking flesh firmness and soluble solid content.

### Irrigation Water Productivity and Soil Water Stress Coefficients

For each orchard management, irrigation water productivity (WP_I_) and soil water stress coefficient (Ks) were calculated.

(2) $$WP_{I}(kg\;m^{-3})=\frac{Y(kg\cdot m^{-2})}{I(m^{3}\cdot m^{-2})}$$

Where Y is yield of marketable product and I is seasonal irrigation volume. WP_I_ (Fernández et al., 2020) stands for the kilograms of marketable peaches produced per unit of irrigation water (m^3^) supplied to the orchard.

Ks (dimensionless) represents a transpiration reduction factor depending on available soil water ranging from 0—maximum stress, to 1—no stress. When SWC is below the RAW threshold, Ks values are less than 1 and crops activate physiological mechanisms (stomatal closure) to reduce transpiration. Daily Ks values (Ks_i_) were calculated setting 0.5 the “p” value, *i.e.* the readily available water threshold (Allen et al., 1998):

(3) $$Ks_{i}=\frac{Wc_{i}-fc}{p(fc-wp)-wp}$$

Seasonal Ks values were standardized (Ks_st_) with minimum values (Ks_min_) calculated during the irrigation season:

(4) $$Ks_{st}=\frac{\left(\frac{\Sigma Ks_{i}}{n}\right)}{Ks_{min}}$$

where n is the number of Ks_i_ occurring during the irrigation season.

### Statistical Analysis

Ks_st_ was analyzed considering a three-way ANOVA (Lr, Sm, and Ir) by season (2017 and 2018) using the statistical package Statgraphics Plus 5.1 (StatPoint Technologies Inc., Warrenton, VA). Duncan’s multiple range *post-hoc* test was used for mean separation when the interaction between three factors indicated significant differences.

In order to manage the possible influence of undesired differences of crop load on fruit growth, yield and WP_I_, the covariance analysis (ANCOVA) was performed, considering the number of fruits as the covariate variable.

The relationship between Ks_st_ and yield was performed by the regression analysis (through Statgraphics Plus 5.1).

## Results

### Agrometeorological Conditions

**Figure 3:**
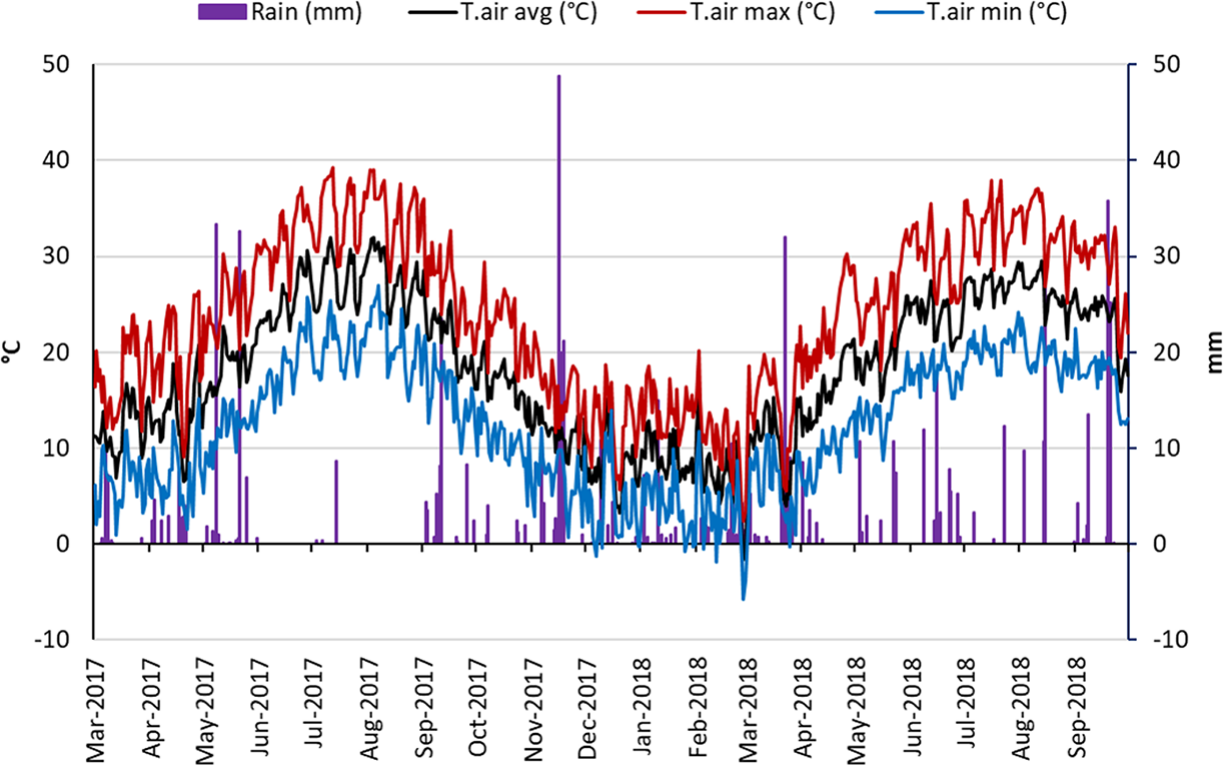
Trend of air temperature (min, max, and avg) and rain during two experimental seasons (2017–2018).

The two studied seasons (1^st^ April–30^th^ September) were similar for air temperature (average temperature 22.3°C and 22.8°C, in 2017 and 2018 respectively). Temperatures ranged between 38°C in summer and rare events below 0°C. The total rainfall was higher in 2018 than in 2017; during the irrigation period (1^st^ June–10^th^ September) the extent of the difference between the two seasons was 133 mm (**Figure 3**).

**Figure 4:**
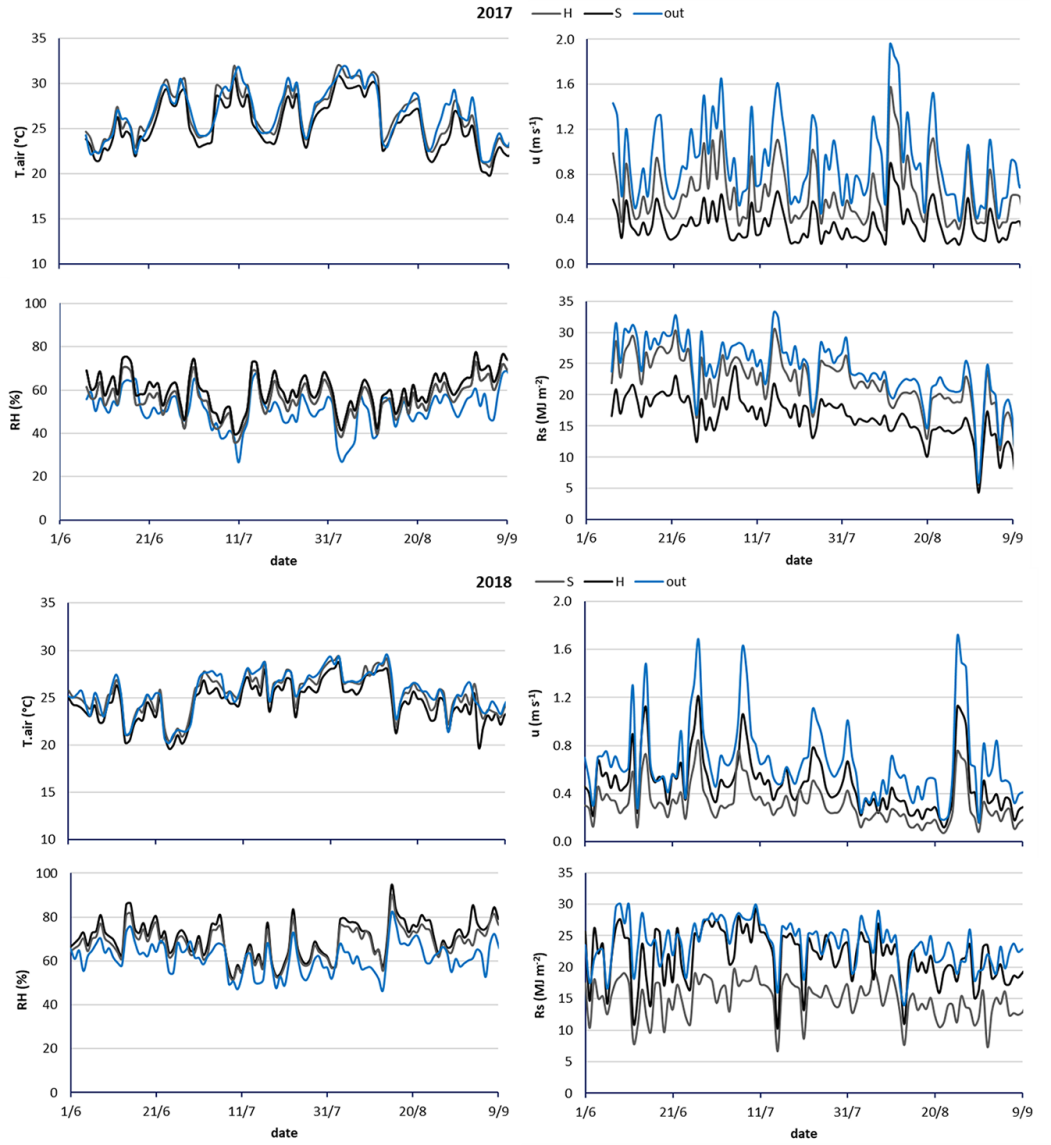
Trend of daily agrometeorological data (T. air, air temperature; u, wind speed; RH, air relative humidity; Rs, solar radiation) measured from the weather station (out), under Hail (H) and Shading hail (S) nets during two seasons (2017 and 2018).

Light modulation by means of hail net or shading hail net changed the microclimate of the orchard. **Figure 4** shows the evolution in time of four agrometeorological variables (Tair, RH, u, and Rs) detected under the light shelters (H and S) and from the nearby weather station. The effects on air temperature due to the two covering materials were similar for the two seasons. The hail net affected negligibly the temperature with an average reduction of 0.2°C, while the shading hail net reduced the air temperature by 1.1°C (**Table 3**). With respect to outside values, air relative humidity increased by 9.4 and 14% in hail and shade nets, respectively. Coverage also led to a windbreak effect on the peach orchard, reducing the wind speed by 30 and 57.5% on average for hail and shading hail net respectively. This effect was more evident in 2017, due to the greater windiness characterizing the season (0.93 ms^−1^ in 2017 and 0.65 ms^−1^ in 2018). The reduction in solar radiation was in line with the net manufacturer specifications: 9% for the hail net and 32% for the shading hail net (**Table 3**).

**Table 3:** – Average values (avg) of four agrometeorological parameters (T: air temperature in °C; RH air relative humidity in %; u: wind speed in m s^-1^; Rs: solar radiation in MJ m^-2^) measured out (from the nearby agrometeorological station) and into the peach orchard covered by hail (H) and shade (S) nets.

Parameter	u.m	avg						u.m	V			
		2017			2018				2017		2018	
		Out	H	S	out	H	S		H	S	H	S
T	°C	26.5	26.3	25.3	25.5	25.3	24.4	°C	−0.2	−1.2	−0.2	−1.1
RH	%	51.8	57.0	60.9	61.5	68.0	70.7	%	9.1	15.0	9.7	13.1
u	m s^−1^	0.93	0.63	0.36	0.65	0.47	0.30	%	−31.8	−61.6	−28.0	−53.7
Rs	MJ m^−2^	23.8	21.7	16.2	23.7	21.6	16.1	%	−9.0	−31.9	−8.9	−32.1

Variations (V) between “out” and “into” data are also reported. Values refer to the 2017 and 2018 irrigation seasons (June 1^st^–September 10^th^).

### Soil Water Content

**Figure 5:**
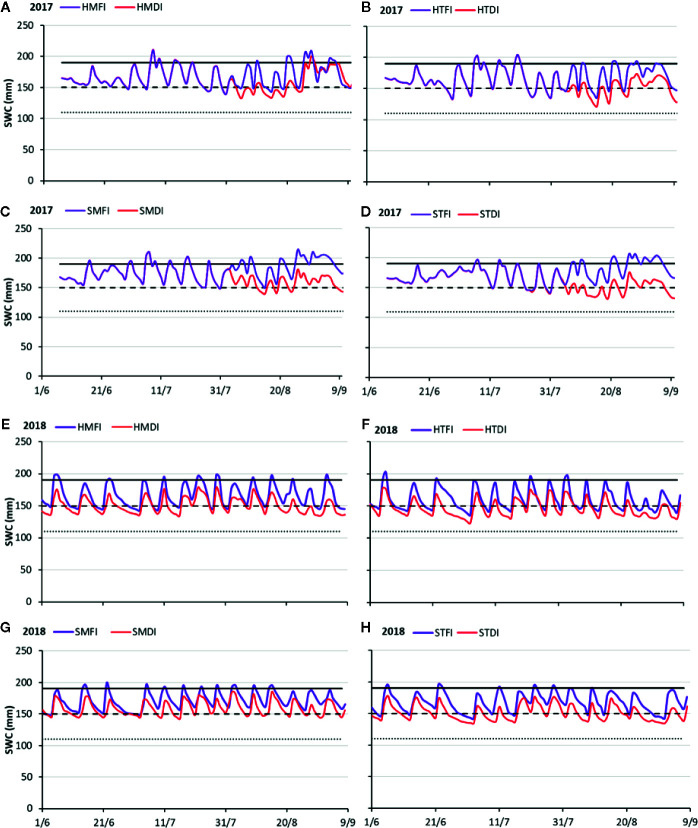
Trends of daily soil water content (SWC) during 2017 **(A–D)** and 2018 **(E–H)** observed on HMFI and HMDI **(A–E)**, HTFI and HTDI **(B–F)**, SMFI and SMDI **(C–G)**, STFI and STDI **(D–H)** crop managements. The values of the field capacity (continuous line), readily available water threshold (dashed line), wilting point (dense dashed line) are also shown.

**Figure 6:**
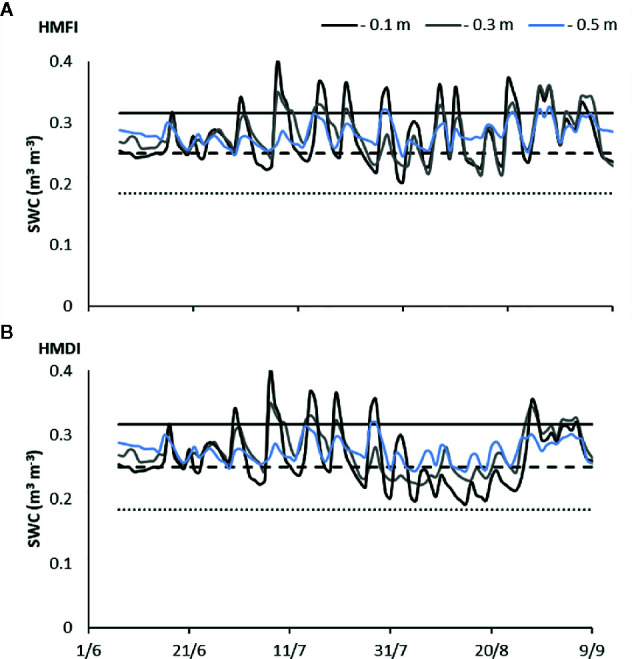
Daily variations of soil water content (SWC) during 2017 season at three depths (−0.1, −0.3 and −0.5 m) for HMFI **(A)** and HMDI **(B)** managements. The values of the field capacity (continuous line), readily available water threshold (dashed line), wilting point (dense dashed line) are also shown.

The irrigation scheduling in the FI treatments allowed for optimizing the SWC within the RAW threshold (150 mm), avoiding any water stress. In both seasons, the SWC (from −0.10 m to −0.50 m soil depth) reached the field capacity, after irrigation or consistent precipitations. Only at the end of the 2017 irrigation season, SWC values exceed field capacity. At the beginning of July 2018, irrigation was skipped due to technical problems: in this period, a reduction in SWC under RAW was observed also in FI treatments (**Figure 5**). **Figure 6** shows the trend of daily SWC monitored during 2017 season at three depths (−0.1, −0.3, and −0.5 m), for both irrigation treatments (FI and DI), under hail nets (H) and mulching (M) treatments. The SWC in the top layer (−0.1 m) showed higher variations as an effect of irrigation and soil evaporation. Instead, at −0.3 m SWC variations were smaller and in the deepest layer (−0.5 m) they can be retained negligible. Starting from August, under DI treatment, the SWC in the top layer (−0.1 m) varied between the RAW threshold and the wilting point, while it was close to RAW threshold at deeper soil layers.

In 2017, the SWC (mm) pattern along the whole soil profile for the eight treatments was quite similar (**Figure 5**), and SWC variations were dependent on irrigation regime (FI and DI). SWC in the FI treatments ranged between field capacity and RAW threshold. SWC values decreased under RAW threshold, just before irrigations in the crop management with hail nets (HTFI and HMFI), while they were always above the RAW threshold in the shading hail net treatments. In August, DI treatment showed few periods when SWC was below the RAW threshold (**Figure 5**). However, it occurred less frequently in SMDI than in the other DI treatments.

In 2018, SWC observed in the FI treatment varied between the field capacity and RAW threshold. Just before the irrigation supply SWC dropped below RAW in orchard plots under the hail nets; while SWC kept above the RAW threshold in the shading hail net treatments. As for the DI treatments, SWC values above the RAW threshold occurred only in the SMDI treatment (**Figure 5G**).

### Daily and Seasonal Soil Water Stress Coefficients

In this section the daily dynamics of soil water stress coefficient (Ks) is analyzed and a seasonal value is conceptualized, defined as standardized soil water stress coefficient (Ks_st_).

**Figure 7:**
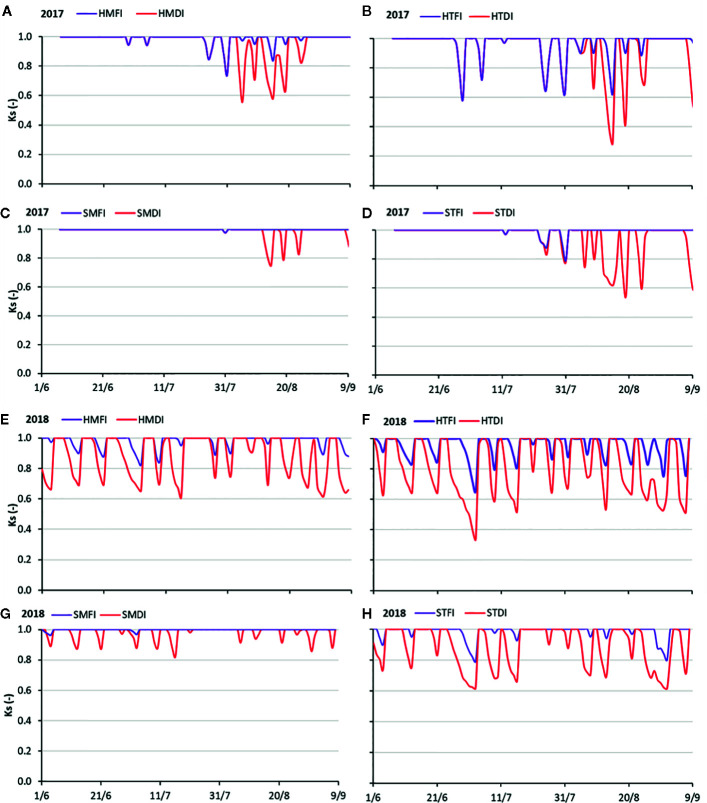
Trends of daily soil water stress coefficients (Ks) during 2017 **(A–D)** and 2018 **(E–H)** observed on HMFI and HMDI **(A–E)**, HTFI and HTDI **(B–F)**, SMFI and SMDI **(C–G)**, STFI and STDI **(D–H)** crop managements.

In 2017, the combination of hail net and soil tillage (H, T) resulted in a greater occurrence of Ks values lower than 1. The minimum Ks values attained 0.6 for fully irrigated treatment (HTFI) and 0.3 for HTDI (**Figure 7B**). The presence of mulching improved the SWC, and consequently, only Ks values higher than 0.75 were occurring in the case of FI treatment. While under the DI treatment, Ks lowered to 0.6 (**Figure 7A**).

As for the T soil management, Ks was always higher than 0.8 when peach orchard was fully watered (FI), while it became low (around 0.6) in the DI treatment (**Figure 7D**). When mulching was associated with shading hail net and full irrigation (SMFI) Ks attained the maximum value (1) during the whole irrigation season. In the case of the deficit irrigation (SMDI), seldom (only thrice) the Ks values reduced to 0.8 during the irrigation season (**Figure 7C**).

In general, DI treatments in 2018 showed Ks values lower than FI. As observed in 2017, also in the 2018 season the most favorable soil water conditions occurred by combining shading hail net (S) and mulching (M). Under FI management, no water stress in the soil was observed, and Ks values were close to 1 all the season long. Under DI treatment (SMDI), Ks never dropped below 0.82 (**Figure 7G**). The combination of hail net (H) and soil tillage (T) determined the worst soil water conditions (**Figure 7F**) in FI (HTFI) and in DI irrigation regime (HTDI).

The 2017 season was hotter, drier, and windier than the 2018 one. Since the weather trends affected significantly the peach orchard behavior, the Ks values are analyzed separately in 2017 and 2018 seasons. Due to the peculiar meteorological conditions, Ks_st_ in 2017 was lower than in 2018 (**Table 4**).

**Table 4:** Effects of Light regimes (Lr), Soil managements (Sm), Irrigation regimes (Ir) on Standardized Soil Water Stress Coefficient (Ks_st_) in 2017 and 2018 seasons and their interactions (p).

Treatments		Ks_st_			
		2017		2018	
Light regime (Lr)	H	0.56	b^2^	0.66	b
	S	0.78	a	0.83	a
Soil manag. (Sm)	T	0.58	b	0.65	b
	M	0.77	a	0.83	a
Irr. regime (Ir)	FI	0.78	a	0.82	a
	DI	0.56	b	0.66	b
P^1^	Lr	0.0000	***	0.0000	***
	Sm	0.0000	***	0.0000	***
	Ir	0.0000	***	0.0000	***
	Lr-Sm	0.2660	ns	0.0535	ns
	Lr-Ir	0.4234	ns	0.0063	**
	Sm-Ir	0.0078	**	0.1227	ns
	Lr-Sm-Ir	0.0036	**	0.0294	*

^1^*, **, *** and ns denote statistical significance at the 0.05, 0.01 and 0.001 levels and the absence of significance, respectively.

^2^Different letters in the columns indicate significant differences between treatments within the same factor, according to the Duncan’s test (P < 0.05).

During the 2018 irrigation season rainfall was higher and lower the vapor pressure deficit (VPD), calculated from the average values of agrometeorological data (reported in **Table 3**). The VPD seasonal values were 1.67 in 2017 and 1.26 kPa in 2018. As a consequence, the evapotranspirative demand (and soil dehydration also) in 2017 was higher than in 2018.

The statistical analysis revealed that Lr, Sm, and Ir factors affected in the same way Ks_st_ in both seasons and that the highest Ks_st_ values corresponded to the S, M, and FI treatments (**Table 4**). In both seasons S, M, and FI significantly raised the SWC and consistently Ks_st_ resulted in an increase by 24%, with respect to H, T, and DI. It should be underlined that the increase extent was the same for the three considered factors (Lr, Sm, and Ir).

**Figure 8:**
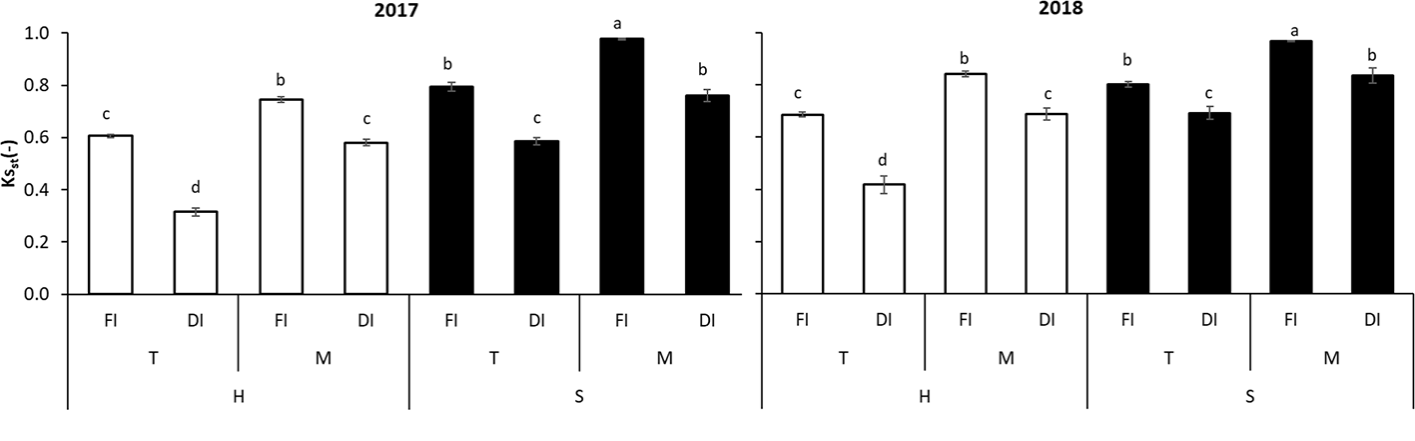
Combined effects of Light regimes (H and S), Soil managements (T and M), Irrigation regimes (FI and DI) on standardized soil water stress coefficient (Ks_st_) for each season (2017 and 2018). Different letters indicate significant differences according to the Duncan’s test (P < 0.05).

By analyzing the significant interactions among all factors, the same ranking was derived in both seasons (**Figure 8**) and four crop management groups appeared: 1) SMFI (Ks_st_ = 0.98–0.97); 2) SMDI–STFI–HMFI (Ks_st_ = 0.76–0.84); 3) STDI–HMDI–HTFI (Ks_st_ = 0.59–0.69); 4) HTDI (Ks_st_ = 0.31–0.42).

Among the DI treatments, SMDI showed the highest Ks_st_ values (0.80 average value on two seasons), consistent with those observed in the full irrigated (HMFI, STFI) treatments (**Figure 8**). This value was 19% higher than the control treatment (0.65 in HTFI).

### Fruit Growth, Yield, and WP_I_

The influence of fruit number was significant for Yield and WP_I_ in both years while it did not affect fruit growth (Volume and AGR) and the final fruit weight.

**Figure 9:**
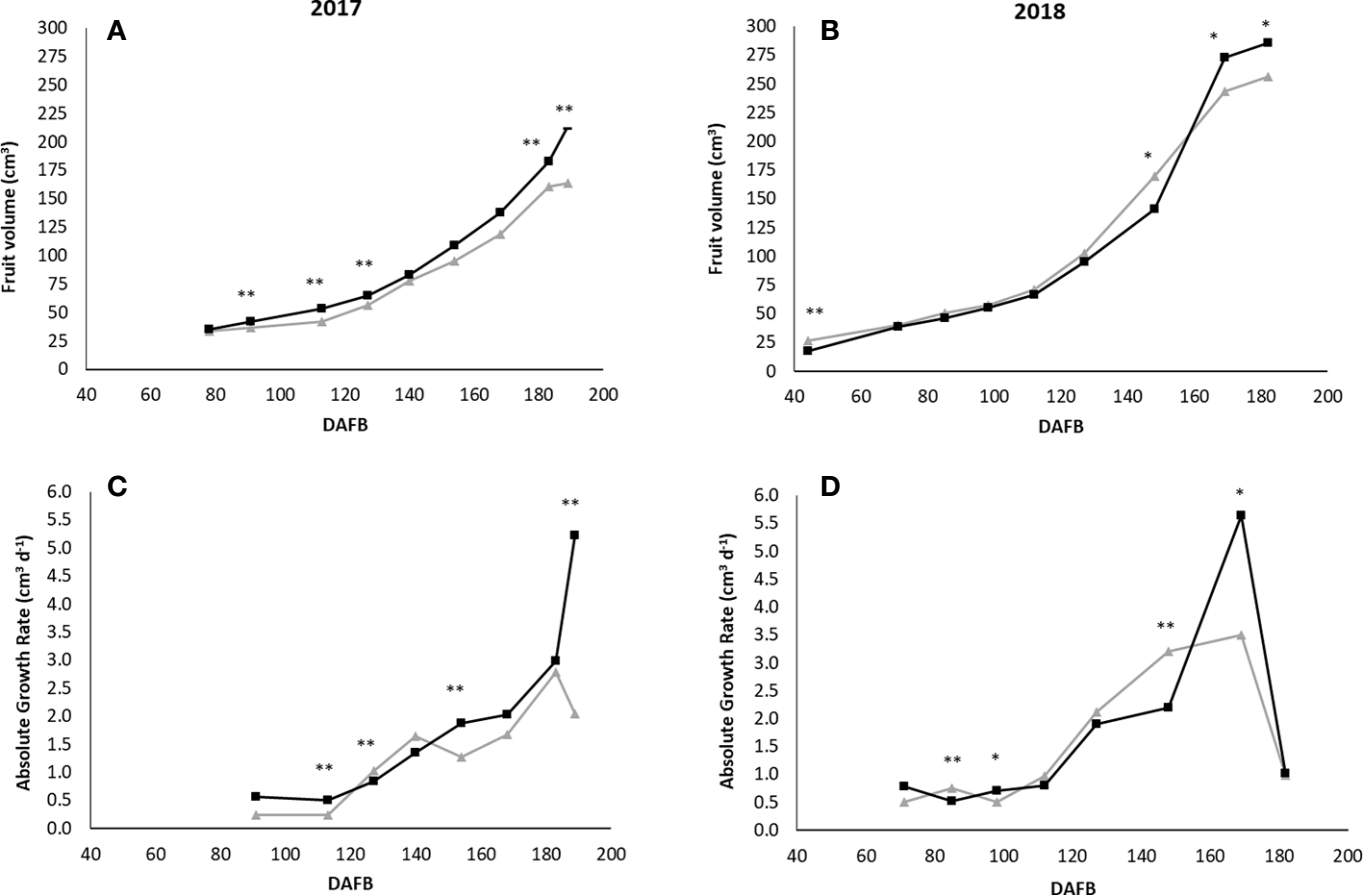
Fruit volume (cm^3^; **A**, **B**) and Absolute Growth Rate (AGR, cm^3^ d^−1^; **C**, **D**) under Light regimes (H, gray line; S, black line) during fruit development (DAFB, days after full bloom) in 2017 and 2018. ** and * indicate a significant difference at P < 0.01 and P < 0.05, respectively.

In 2017 and 2018 the full bloom occurred on March 7^th^ and March 26^th^, respectively. In both years, the interaction among the three factors was not significant for fruit growth; fruit volume and AGR were affected by Lr. In 2017, at the beginning of the fruit monitoring survey (78 DAFB; days after full bloom), S and H treatments showed similar fruit volume and AGR. Excluding on 140 and 154 DAFB, S showed the fruit volume higher than H for all the season long (**Figure 9A**). The same behavior was observed for AGR, and the statistical difference between S and H occurred on 91, 113, 154, and 189 DAFB (**Figure 9C**). In 2018, at the beginning of monitoring (44 DAFB), fruit in H were bigger than in S (26.7 cm^3^ and 17.4 cm^3^, respectively). This difference disappeared till 127 DAFB. On 148 DAFB, H showed fruit volume higher than S, while on 169 DAFB an opposite behavior was observed. This difference was maintained until harvest when the fruit volumes were 286.1 cm^3^ and 256.3 cm^3^ in S and H, respectively (**Figure 9B**). AGR pattern followed the same trend. Moving from 148 to 169 DAFB, AGR in S varied from 2.19 cm^3^ d^−1^ to 5.64 cm^3^ d^−1^, while in H it changed from 3.20 cm^3^ d^−1^ to 3.50 cm^3^ d^−1^, resulting higher in S than in H (**Figure 9D**).

**Figure 10:**
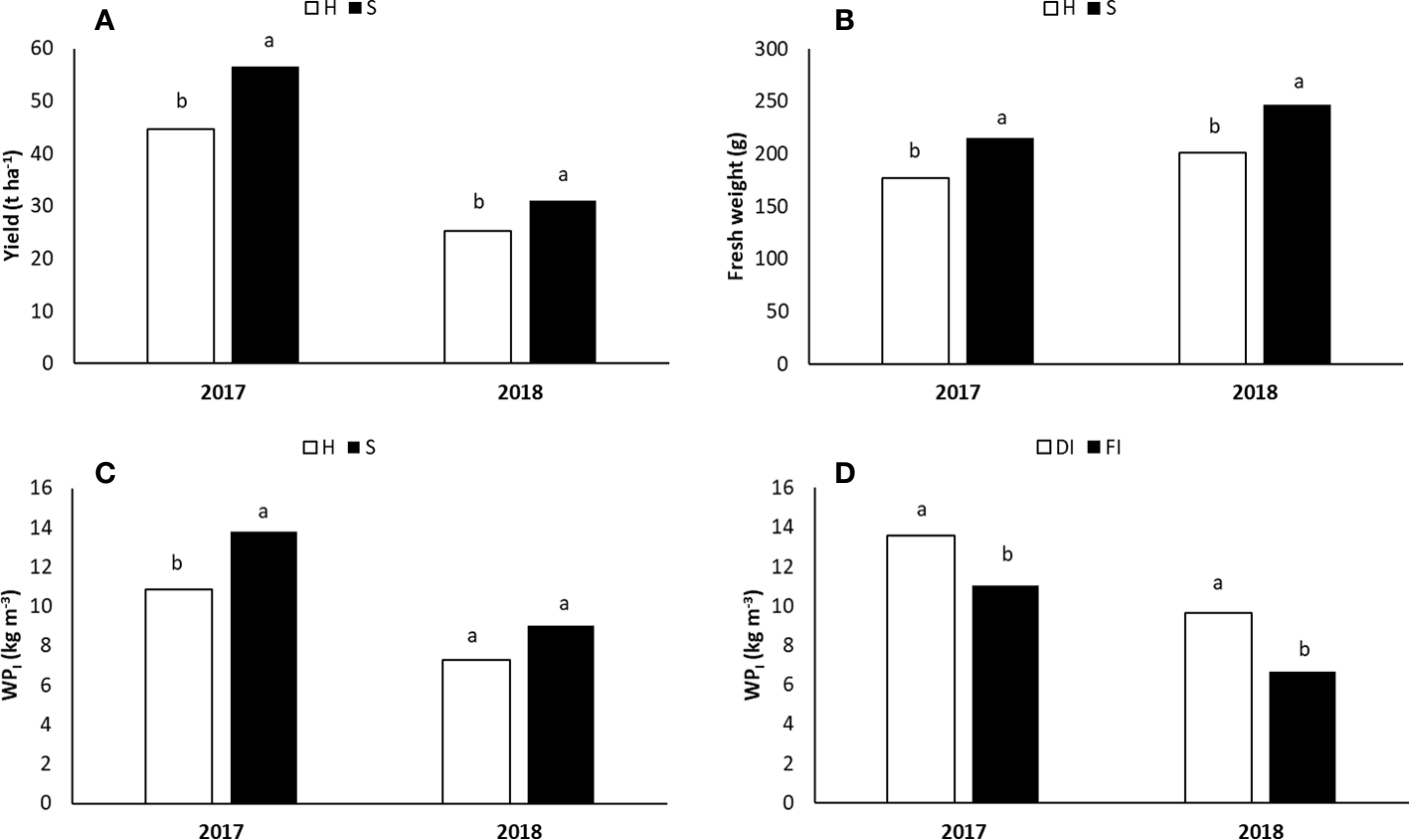
Yield (t ha^−1^; **A**) and fresh weight (g; **B**) under the two light regimes (Shading hail net *vs* Hail net); Irrigation Water Productivity (WP_I_, kg m^−3^) under the two light regimes **(C)** and the two water regimes (Deficit Irrigation *vs* Full Irrigation; **D**) in 2017 and 2018. Different letters indicate a significant difference at P < 0.05.

In both years, the interaction among the three factors was not significant for yield, fruit weight, and WP_I_. With respect to the H treatment, yield and average fruit weight were significantly higher than S in 2017 and in 2018 (**Figures 10A, B**), while no difference for fruit flesh firmness and solid soluble content was recorded (data no showed). In the latter season, yield decreased by 55% if compared to 2017 data. Such reduction was due to the low temperatures that occurred during the flowering period (March 2018) which led to freezing conditions and, in turn, to flower drop. In both years (**Figure 10A**), yield was significantly higher under shading hail net than under hail net: 56.6 t ha^−1^ *vs* 44.7 t ha^−1^ in 2017, and 31.0 t ha^−1^ *vs* 25.2 t ha^−1^ in 2018.

Water productivity (WP_I_) was significantly affected by the irrigation regime (DI *vs* FI) for both years (**Figure 10D**). However, in 2017 a significant difference due to the Lr on WP_I_ was also recorded (**Figure 10C**). In particular, in 2017 WP_I_ was significantly higher in DI (13.57 kg m^−3^) than FI (11.06 kg m^−3^) while in 2018 WP_I_ was 9.62 and 6.66 kg m^−3^ in DI and FI, respectively. In 2017 WP_I_ was higher for S (13.77 kg m^−3^) than under the H (10.86 kg m^−3^) roofing.

## Discussion

There are no data showing the effects of covering the peach orchard with mulching, so the results of this study represent a novelty in this area. The agronomic strategies aiming at modulating the light (Lr) or at managing the soil (Sm) or at reducing irrigation volume (Ir) acted on SWC. In the peach orchard, when the DI technique was applied, actually SWC reduced (**Figures 5** and **6**) and soil water stress arises (**Figure 7**). However, the number of the stress events along the vegetative cycle and their intensity changed consistently with the agro-techniques. The Ks_st_ has been here proposed as a synthetic indicator for quantifying the soil stress dynamics during the peach season. The three agronomic factors (Lr, Ir, and Sm) did affect significantly the Ks_st_ values detected in both years (**Table 4**). Moreover, the significant interactions on both years (**Table 4**) suggested that the irrigation regime alone can’t explain the moisture conditions in the soil. The role in determining the soil stress on the crop was shared by the irrigation regime and the other agronomic management practices (Sm and Lr). As evidence, the Ks_st_ values in the limited irrigation treatments (DI) were reduced when these techniques were combined together. The Ks_st_ could be retained as the link between the changes in the SWC and the crop behavior.

Annual and perennial crops (Wery, 2005), if affected by soil water deficit, react whenever a soil water deficit arises and the SWC drops below the RAW threshold (Foreya et al., 2016). In theory crops from DI treatments, despite their experience of soil stress periods when SWC drops below RAW, should not show significant lower fruit growth and final yield, and it is confirmed also in this study. Such evidence, if analyzed in the light of the significant interaction of the three agronomic factors on Ks_st_ in both seasons, suggests that the DI should be coupled with those agronomic strategies which preserve the water stored in the soil profile to increase its effectiveness.

Shading hail net used in this study reduced direct incident solar radiation by 32% and the microclimatic conditions in the peach orchard changed (**Table 3**). The modified microclimatic conditions indirectly improved SWC in the peach orchard as revealed by the Ks_st_ values (0.8 being the average value from the S managements). The reduction of 9% of the incident radiation showed the lowest values of Ks_st_, especially when it was combined with the deficit irrigation technique (H–DI-treatment average Ks_st_ value = 0.61).

The SWC worsened when mulching was not applied and irrigation was deficient (HTDI, Ks_st_ value = 0.37). On the contrary, techniques aiming at reducing soil evaporation ameliorated SWC. When mulching is adopted, in comparison with the tilled treatments, Ks_st_ generally increased: 0.62 and 0.8 in T and M treatments, respectively. With respect to tillage management, in mulching treatment Ks_st_ increased by 31% and production by 12% (**Figure 8**). A similar trend was reported by Wang H. et al. (2015) in a Chinese semi-arid environment, where mulching led to increase the SWC by 14% and yield production by 28%, with respect to the traditional soil tillage.

Ks_st_ increased by 21, 73, 21, and 25% moving from HTFI to HMFI, from HTDI to HMDI, from STFI to SMFI, and from STDI to SMDI, respectively (**Figure 8**). This result emphasizes that mulching positively affects the soil water status (Wang H. et al., 2015; Wang C. et al., 2015). Further improvement was reached when other techniques aimed at reducing soil evaporation were adopted conjointly. Mulching together with shading hail net further increased Ks_st_, and the maximum value (0.97 in SMFI) was reached when evapotranspiration was mitigated by reducing irradiance and soil evaporation. Under shading hail net, saving water by means of DI demonstrated to be a suitable tool: the Ks_st_ in the SMDI treatment was 0.80, of the same order than that observed, in average, in all the FI treatments (**Figure 8**).

The moderate reduction (about 30%) of incident solar radiation improved peach yield and fruit weight (**Figures 10A, B**). Microclimate as well as SWC was more favorable to peach carbon assimilation. Previous studies on peach and apple showed that under moderate shading, incident radiation and leaf temperature attained optimal range for photosynthesis and the photoinhibition risk was alleviated. As a consequence, carbohydrate allocation to fruit was more abundant and less photoassimilates were used for photoprotection and recovery (Losciale et al., 2011b; Lopez et al., 2018). Fruit growth and fruit weight were higher under shading hail net than in H (**Figures 9** and **10**). Recent studies showed that very low, as well as very high, VPD values could be limiting fruit growth. The former, because it reduces fruit skin transpiration (the driving force for fruit growth) and the latter, because it can generate an imbalance between the incoming and outcoming water at the fruit scale (Morandi et al., 2010).

Water productivity (WP_I_) stands for the ability of a crop system to convert the irrigation water in marketable fruits. WP_I_ was affected by the irrigation strategy as well as by the Lr when the crop load was not reduced (**Figures 10C, D**). DI ameliorated WP_I_ by 20 and 40% in comparison to FI, in 2017 and 2018, respectively. Moreover, in 2017 water productivity from the S treatments was about 20% higher than that observed from H (**Figures 10C, D**).

**Figure 11:**
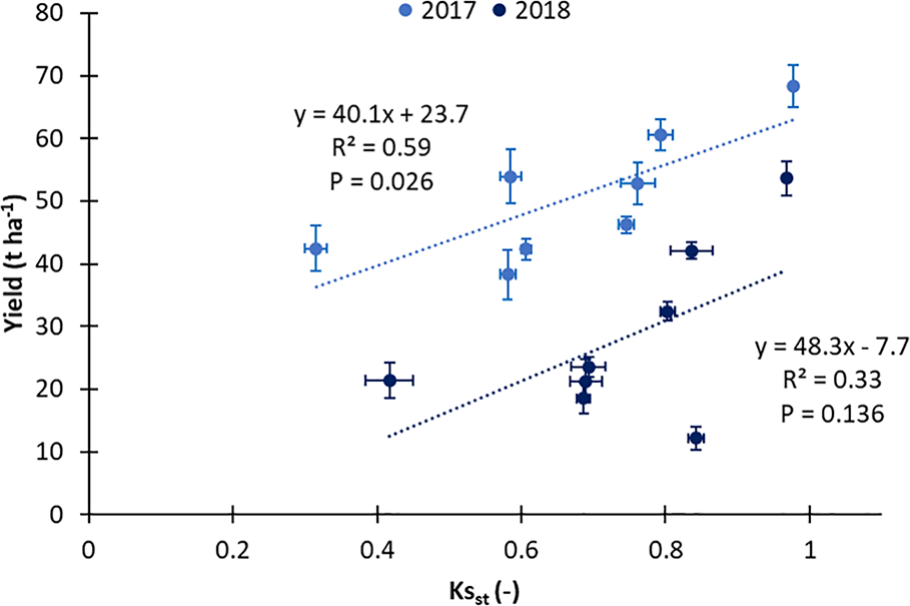
Relationship between yield and standardized soil water stress coefficient (Ks_st_) calculated for all treatments in two seasons (2017 and 2018).

The best water friendly management was SMDI, as it showed Ks_st_ and yield, in line with the most productive treatments (S), and its WP_I_ was one of the highest (S, DI) values reported in the present study (**Table 4**, **Figures 10C, D**). Furthermore, in 2017 the significant linear relationship between yield and stress coefficient (**Figure 11**) makes it reliable to retain Ks_st_ as an appropriate index to identify the water stress and the environmental (VPD in 2017 was higher than 2018) conditions well-suited for peach cultivation. The peculiar meteorological trend in 2018 (probably also because the late frost altered the final yield values) did not allow for holding the same inference (**Figure 11**) also for a second year (when the linear relationship was not statistically significant), suggesting that the final yield was the effect of concurrent factors, and Ks_st_ index needs to be refined taking into consideration additional information, as crop load (Morandi et al., 2011) or the potential yield of the species.

These results emphasize that the detrimental effect of water shortage could be balanced by suitable modulation of the microclimate and contrasting the extra water losses (Losciale et al., 2011; Lopez et al., 2018; Zheng et al., 2019). Recent research suggested that deficit irrigation (75% ETc) can improve WP_I_ with a minimal (less than 10%) reduction in yield (Zhou et al., 2017). The combination of three “water friendly” techniques, evaluated in the present study, increased yield by 25% and improved WP_I_ by 20–40% (considering hail net covered and full irrigated treatments as control). The results here reported suggest the positive effect of the integration of different agro-techniques as a promising (from an ecological and economic point of view) strategy for saving irrigation water.

## Conclusions

Since the sustainability of full irrigation techniques is questionable in the Mediterranean climate environments, alternative approaches should be proposed. In the case of peach trees, mainly the late ripening varieties, irrigation management aiming at provoking temporary soil water deficit periods can improve WP_I_ but can trigger yield decrease. The use of sound agro-techniques as shading hail net and soil mulching, better if used conjointly, demonstrated to be effective. Through adequate crop managements, also under the Mediterranean climate, it is possible to reduce irrigation water volumes and to improve water productivity (in terms of seasonal irrigation volume).

Outcomes here shown on microclimate modulation and on orchard floor management open new ways to further research aiming at evaluating the response of plant (in terms of physiology, fruit yield and quality) to combined water friendly techniques.

## Data Availability Statement

The raw data supporting the conclusions of this article will be made available by the authors, without undue reservation.

## Author Contributions

PC: conceptualization, methodology, writing original draft. LG: data curation, validation, discussion of productive results. MM: writing, review and editing. PL: definition of experimental setup, formal analysis.

## Conflict of Interest

The authors declare that the research was conducted in the absence of any commercial or financial relationships that could be construed as a potential conflict of interest.
